# Spatiotemporal variation of ringed seal blubber cortisol levels in the Canadian Arctic

**DOI:** 10.1093/jmammal/gyac047

**Published:** 2022-06-17

**Authors:** Wesley R Ogloff, Randi A Anderson, David J Yurkowski, Cassandra D Debets, W Gary Anderson, Steven H Ferguson

**Affiliations:** Freshwater Institute, Fisheries and Oceans Canada, 501 University Crescent, Winnipeg, MB R3T 2N6, Canada; Office of the Minister of Natural Resources Canada, Ottawa, ON K1A 0E4, Canada; Freshwater Institute, Fisheries and Oceans Canada, 501 University Crescent, Winnipeg, MB R3T 2N6, Canada; Department of Biological Sciences, University of Manitoba, 66 Chancellors Circle, Winnipeg, MB R3T 2N2, Canada; Department of Biological Sciences, University of Manitoba, 66 Chancellors Circle, Winnipeg, MB R3T 2N2, Canada; Department of Biological Sciences, University of Manitoba, 66 Chancellors Circle, Winnipeg, MB R3T 2N2, Canada; Freshwater Institute, Fisheries and Oceans Canada, 501 University Crescent, Winnipeg, MB R3T 2N6, Canada; Department of Biological Sciences, University of Manitoba, 66 Chancellors Circle, Winnipeg, MB R3T 2N2, Canada

**Keywords:** Arctic, Arviat, blubber, body condition, chronic stress, cortisol, *Pusa hispida*, ringed seal, stable isotopes, Ulukhaktok

## Abstract

Climate change in the Arctic has widespread and complex effects on the health of animals and their populations. We used radioimmunoassay to measure blubber cortisol in ringed seals (*Pusa hispida*) sampled in Ulukhaktok, Inuvialuit Settlement Region, Northwest Territories, Canada (spring, 2002, 2004–2005, 2007–2012) and Arviat, Nunavut, Canada (autumn, 2003–2012) to examine chronic stress relative to biology (age, sex, length), body condition (blubber depth), and diet (δ^13^C, δ^15^N, and isotopic niche size). Ulukhaktok ringed seals had higher cortisol concentrations overall (0.46 ± 0.04 ng/g) than Arviat ringed seals (0.36 ± 0.03 ng/g), and these higher concentrations were associated with higher muscle δ^15^N and lower blubber thickness. In contrast, blubber cortisol concentrations for Arviat ringed seals decreased with blubber depth and increased with age, though testing of age effects individually suggests that age-related patterns are weak. Annual mean cortisol concentration increased from 2003 to 2012 in Arviat ringed seals, but low sample sizes precluded analysis of annual patterns for Ulukhaktok ringed seals. The trend of increased cortisol over time in Arviat ringed seals suggests that they might be experiencing greater chronic stress over time, which could have implications for numerous population health metrics including reproduction and pup recruitment.

Climate change in the Arctic has led to a reduction in the quantity and quality of sea ice, a key habitat component for numerous species ranging from algae- and ice-associated invertebrates to polar bears (*Ursus maritimus*; Laidre et al. 2015; Hamilton et al. 2017; Hop et al. 2020; Hwang et al. 2020). This reduction in sea ice has allowed for the expansion of human impacts, including increased shipping and industrial activities, exposing animals to an increased risk of contaminant exposure, ship strikes, and noise pollution (Ghosh and Rubly 2015; Hauser et al. 2018; McWhinnie et al. 2018). Higher air and sea-surface temperatures introduce physiological challenges that could lead to hyperthermia in extreme cases and, more commonly, facilitate the northward shift of temperate and subarctic species (Evans and Bjørge 2013; Pinsky et al. 2013; Pecl et al. 2017), including pathogens (Cohen et al. 2018). Competition for food and habitat can arise as southern and marginal species become more common in Arctic environments, impacting the food web at multiple levels (Kortsch et al. 2015; Pecl et al. 2017; Yurkowski et al. 2018). Foraging may become more energetically expensive as habitat becomes more fragmented, and energetic return may be limited for predators that must switch to prey that is more limited in quantity, accessibility, or energetic quality. These and other climate-driven changes will likely present new and compounding challenges for ice-associated Arctic species, which can then lead to population- and ecosystem-wide consequences over time, such as reduced population abundances and impacts on biodiversity (Kovacs et al. 2011; Laidre et al. 2015).

In response to challenging or threatening conditions, animals might experience a stress response, with the release of cortisol and other glucocorticoids facilitating a shift in energy balance to maintain survival or homeostasis, a process that can be maladaptive when stress is chronic (Sapolsky et al. 2000; Romero and Butler 2007; Busch and Hayward 2009). Among the main drivers of chronic stress in animals are changes to habitat and food availability, both of which are threats in the warming Arctic (Kovacs and Lydersen 2008; Doney et al. 2012; IPCC 2014) and have implications for the variation of individual- and population-level responses to environmental change. Glucocorticoid concentration may be used as an indicator of stress, ideally when experimentally validated or paired with other measures of condition (Romero and Butler 2007; MacDougall-Shackleton et al. 2019). Indicators of stress at the individual level can then be used to infer animal health (Atkinson et al. 2015), which, given adequate sample sizes, can indicate the general health of the population. Acute levels of cortisol can be measured in tissues with fast turnover times, such as blood (e.g., Ortiz et al. 2001; DeRango et al. 2019), making these tissues ideal candidates for examining the effects of animal handling during tagging or medical procedures or of the postweaning stress levels of seal pups (Ortiz et al. 2001; Champagne et al. 2012b; Atkinson et al. 2015; DeRango et al. 2019). In contrast, by measuring cortisol in metabolically inert or slow-turnover tissues (e.g., hair, whisker, blubber, and skin; Kershaw and Hall 2016; Bechshoft et al. 2020; Keogh et al. 2020a, 2020b), researchers can get an indication of chronic stress (Atkinson et al. 2015). Chronic stress can cause immunosuppression, altered fight-or-flight responses, lowered body condition, and reduced reproduction and/or offspring recruitment, which have implications for long-term fitness and population health (Gulland et al. 1999; Burek et al. 2008; Ferguson et al. 2017; Romero and Gormally 2019). Arctic marine mammals, like the ringed seal (*Pusa hispida*), are at particular risk of chronic stress, as their life histories are tightly linked with sea ice (McLaren 1958; Kovacs et al. 2011).

Ringed seals are abundant and circumpolar, making them an ideal candidate species to monitor ecosystem changes in the Arctic (Laidre et al. 2008; Hazen et al. 2019). In the summer open-water period, ringed seals feed intensively to build their blubber layer in preparation for the winter, when resources are more limited (Smith 1987; Freitas et al. 2008; Yurkowski et al. 2016b). In autumn, ringed seals continue to forage and can move long distances to overwintering areas (Harwood et al. 2012; Ogloff et al. 2021)—at this time, their condition is typically at its highest of the year (McLaren 1958; Young and Ferguson 2013). During the winter, foraging strategies shift from pelagic to benthic in some areas (Young and Ferguson 2013), and adult ringed seals establish territories and maintain breathing holes in the sea ice, whereas subadults inhabit pack ice areas or move into open water (McLaren 1958; Kelly et al. 2010; Crawford et al. 2012; Harwood et al. 2015). The final stages of gestation, as well as parturition and lactation, occur in the spring and have considerable energetic costs for females (Ryg et al. 1990; Hammill et al. 1991; Lydersen 1995). Following the nursing and mating period, ringed seals also undergo the energetically expensive process of molting, where they shed and regrow their fur (McLaren 1958). During this time, ringed seals are at their poorest condition of the year and have a markedly higher resting metabolic rate (McLaren 1958; Young and Ferguson 2013; Thometz et al. 2020). At southern latitudes, such as Hudson Bay, Canada, declines in ringed seal reproduction and pup recruitment have been observed between 2003 and 2013 (Ferguson et al. 2017), though the mechanisms of these changes are unknown. The present gaps in our understanding of these changes highlight the importance of long-term health monitoring of ringed seals and other marine mammal populations. Some factors of importance for monitoring include morphometric and condition data, chemical tracers (e.g., stable isotopes, contaminants, trace metals), and biomarkers (e.g., hormones, fatty acids, highly branched isoprenoids).

Here, we examine indicators of chronic stress in ringed seals from two Arctic locations in northern Canada over a period of ~10 years (Arviat: 2003–2012; Ulukhaktok: 2002, 2004–2005, 2007–2012). We measured cortisol concentrations in blubber and tested for relationships with a number of biological and demographic factors. Blubber depth was included in analyses as it is a common a proxy for body condition in marine mammals (Crawford et al. 2015; Ostertag et al. 2018). Stable isotopes (δ^15^N, δ^13^C, and isotopic niche) were included as indicators of long-term foraging, as diet and nutritional state are important indicators of animal health. For example, poor nutritional state has been shown to correlate with higher blubber cortisol concentrations in California sea lions (*Zalophus californianus*; Beaulieu-McCoy et al. 2017). Biological and demographic factors—age, length (as a metric of body size), and sex—were included in analyses due to the roles they play in glucocorticoid production and their influence on the pressures faced by individuals (Dantzer et al. 2014). For instance, northern elephant seal pups (*Mirounga angustirostris*) experience high stress, with associated high cortisol levels, during the postweaning fast (Ortiz et al. 2001); some adult female northern right whales experience higher cortisol levels during pregnancy than during intercalving intervals (Hunt et al. 2017); cortisol measurements of pregnant bottlenose dolphins (*Tursiops truncatus*) increase with time postconception (Steinman et al. 2016); adult and subadult male Weddell seals (*Leptonychotes weddelli*) experience high cortisol levels as a cost of territoriality and competition for mates (Bartsh et al. 1992); smaller, thinner belugas (*Delphinapterus leucas*) have been shown to have higher blubber cortisol concentrations than larger individuals (Loseto et al. 2017); blubber cortisol in Indo-Pacific humpback dolphin (*Sousa chinensis*) calves is positively correlated with body length (Guo et al. 2022); and higher hair cortisol concentrations in polar bears have been associated with smaller, lighter, poorer condition individuals (Macbeth et al. 2012). Ringed seals in the present study were sampled in Arviat in autumn, following the summer foraging period, and in Ulukhaktok in spring, when ringed seals are in their molting phase (McLaren 1958). Specifically, we set out to test (i) which factors relate to ringed seal blubber cortisol levels and (ii) whether there is spatial, seasonal, and long-term variation in blubber cortisol levels of ringed seals.

## Materials and Methods

### Biological sampling

**Fig. 1.**
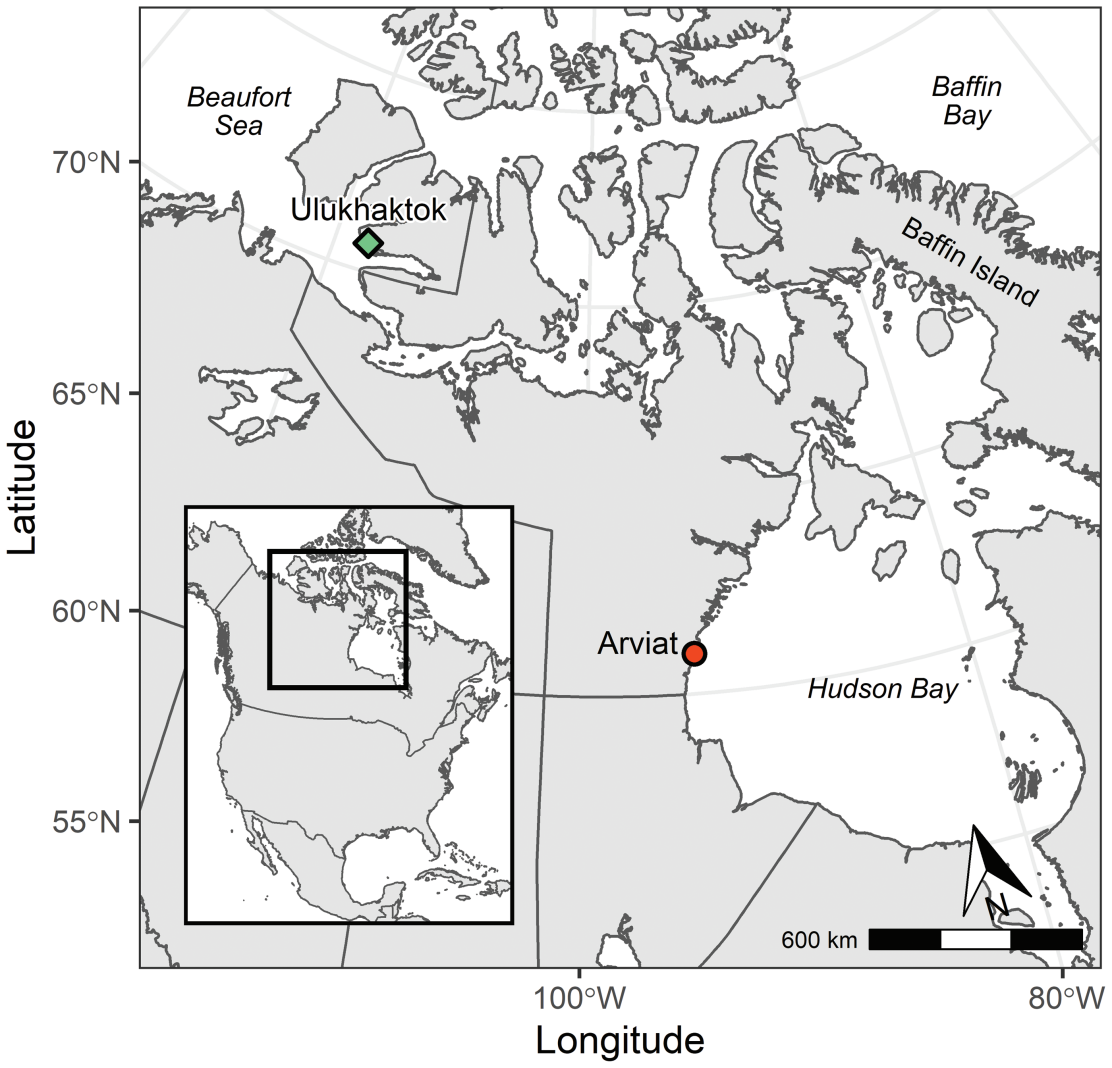
Map highlighting our study regions, Arviat, Nunavut (circle), and Ulukhaktok, Northwest Territories (diamond), within the Canadian Arctic. Ringed seals in the present study were sampled in Arviat in autumn (October and November; 2003–2012) and in Ulukhaktok in spring (May and June; 2002, 2004–2005, 2007–2012). The black square in the inset shows the position of the study area within North America.

Ringed seals were sampled during Inuit subsistence harvests in Arviat, Nunavut, and Ulukhaktok, Northwest Territories, from 2002 to 2012 (Fig. 1). In Ulukhaktok, located within the Inuvialuit Settlement Region, ringed seals were mainly harvested in the spring and summer (May and June; 2002, 2004–2005, 2007–2012), whereas the harvest in Arviat mainly occurred in autumn (October and November; 2003–2012). Ringed seal samples and data from Arviat were also used in Ferguson et al. (2017). Morphometric measurements were taken, and tissues were sampled in field by hunters. Standard length (straight line) was measured as the distance from the tip of the nose to the end of the tail (Committee on Marine Mammals 1967). Samples of blubber—including the hair and skin, through to the outer muscle layer, representing a cross section of all three blubber layers (Strandberg et al. 2008)—and muscle were taken. Lower jaws were collected for age estimation using canine teeth, which were extracted and sectioned to allow counting of the dentin layers. This procedure was conducted in Ulukhaktok or at Matson’s Lab in Manhatten, MT. Ringed seals aged ≥6 years were considered adults, 1–5 years were subadults, and <1 year were pups (Holst et al. 1999; Krafft et al. 2006). Tissue samples were stored at −20°C until they were shipped to the Freshwater Institute in Winnipeg, MB, where they were subsequently stored at −30°C until analysis.

### Cortisol extraction and analysis

We visually assessed blubber sample quality for all samples used in this study, including only those which showed no signs of degradation (e.g., yellowing of the tissue, odor; Trana et al. 2015). We subsampled even, cross-sectional pieces (innermost to outermost, all layers) of tissue from the blubber sample to account for any differences in cortisol concentrations between blubber layers, as they have been shown to differ metabolically and functionally (Strandberg et al. 2008). Samples were then freeze-dried at −50°C for ~48 h. Cortisol extraction and radioimmunoassay were conducted using the procedures outlined in Trana et al. (2015). We determined cortisol extraction efficiency by adding a known volume of tritium-labeled cortisol to a sample, processing along with the others, and measuring the level of radioactivity remaining. Blubber mean cortisol extraction efficiency for this study was 60 ± 4% (*n* = 11). Inter-assay variation was 20 ± 5% (*n* = 23), and intra-assay variation was 6 ± 3% (*n* = 5). Cortisol results are reported as ng/g of blubber tissue.

### Stable isotope analysis

We subsampled ~3–5 g of ringed seal muscle tissue from each muscle sample for stable isotope analysis. Subsamples were freeze-dried at −50°C for 48 h and homogenized using a mortar and pestle. Lipids were extracted using a 2:1 chloroform:methanol solvent mixture, based on the established Bligh and Dyer (1959) method. Dried, homogenized muscle samples were placed into 10-ml centrifuge tubes, and 5 ml of solvent was added. We vortexed samples for ~1 min and allowed them to sit overnight in a 30°C water bath. Samples were then centrifuged for 5 min at 3,000 rpm, and the supernatant was poured off and discarded. We rinsed the remaining pellets with an additional 5 ml of chloroform:methanol, vortexed, and centrifuged again without the overnight soak. This process was repeated, and sample pellets were then dried overnight under a fume hood.

We rehomogenized lipid-extracted muscle tissue and weighed 400–600 μg of tissue into tin capsules. Ratios of ^13^C:^12^C and ^15^N:^14^N were measured using a Thermo Finnigan Delta^Plus^ mass spectrometer equipped with an elemental analyzer at the Great Lakes Institute for Environmental Research, University of Windsor. Ratios of ^13^C:^12^C and ^15^N:^14^N were measured in samples and in Pee Dee Belemnite and atmospheric nitrogen standards, respectively. Relative differences of isotopic ratios (δ), measured in ‰, were determined using the following formula: $\delta^{x}E=\left(\frac{R_{sample}-R_{standard}}{R_{standard}}\right)\;\times \;1,000$, where ^x^*E* refers to ^13^C or ^15^N, and *R* refers to the ratio of heavy to light isotopes of element *E* in either the sample or the standard.

### Body condition

As a proxy for body condition, we measured the depth of the blubber (to the nearest 10th of a cm) on the ventral side of the seal at the sternum using a ruler.

### Statistical analysis

We grouped seals by sampling location for analyses. All groups were checked for normality and homoscedasticity prior to statistical analysis using Shapiro–Wilk tests of normality and Levene’s tests of homogeneity of variance. Cortisol data were highly non-normal, so a log-transformation was applied for subsequent analyses. Outliers in the cortisol data were removed by calculating *Z*-scores and removing values with *Z*-scores ≥ |3|, resulting in omission of three values. We used the SIBER package v2.1.4 (Jackson et al. 2011) in R version 4.1.0 (R Core Team 2019) to calculate isotopic niche sizes (standard ellipse area corrected for small sample sizes; SEA_C_) by location, year, and age-class. The standard ellipse area represents a standard deviation around the bivariate mean and encompasses ~40% of data, considered representative of the core population (Jackson et al. 2011). Yearly SEA_C_ was also non-normal and was log-transformed prior to statistical tests. We used tests of difference to examine age-class (categorical) effects; specifically, we used ANOVA, Kruskal–Wallis rank-sum tests, *t*-tests, or Wilcoxon rank-sum tests—depending on sample sizes, normality, and homoscedasticity—to test for differences in cortisol, condition, and stable isotopes between ringed seal age groups.

Generalized linear mixed models (GLMMs) were run using the lmer function in the lme4 package v1.1-23 (Bates et al. 2015) in R version 4.1.0 (R Core Team 2019) to test for predictive factors of ringed seal blubber cortisol, with model runs on both an annual timescale (to allow for inclusion of annual isotopic niche size and to examine patterns over time) and irrespective of year. When not grouped by year, year was included in the models as a random effect, and age was included as a continuous variable. Models were assessed using Akaike’s information criteria. The inclusion of interaction terms was explored, but, ultimately, stepwise removal of variables during data exploration resulted in all interaction terms being removed early on, with the same end points as when interactions were excluded. For this reason, and to keep analyses simple and concise, interaction terms are not presented here. We assessed multicollinearity in our full models using variance inflation factors (VIFs) calculated via the check_collinearity function in the R package performance v0.8.0 (Lüdecke et al. 2021). For models using data throughout the sampling period, all VIFs were low (≤1.7 for Arviat and <3.2 for Ulukhaktok). For models assessing annual mean values across variables, VIFs for some variables were moderate (8.4 and 6.6 for age-class and length, respectively); however, upon removal of age-class in subsequent model runs, all VIFs were ≤2.7. Model performance for each model was assessed using conditional *R*-squared values (*R*^2^_C_—indicating the variance explained by the entire model, including both fixed and random effects), calculated using the r.squaredGLMM function in the R package MuMIn v1.43.17 (Bartoń 2020). Results are reported as mean ± standard error (*SE*) throughout.

## Results

**Fig. 2.**
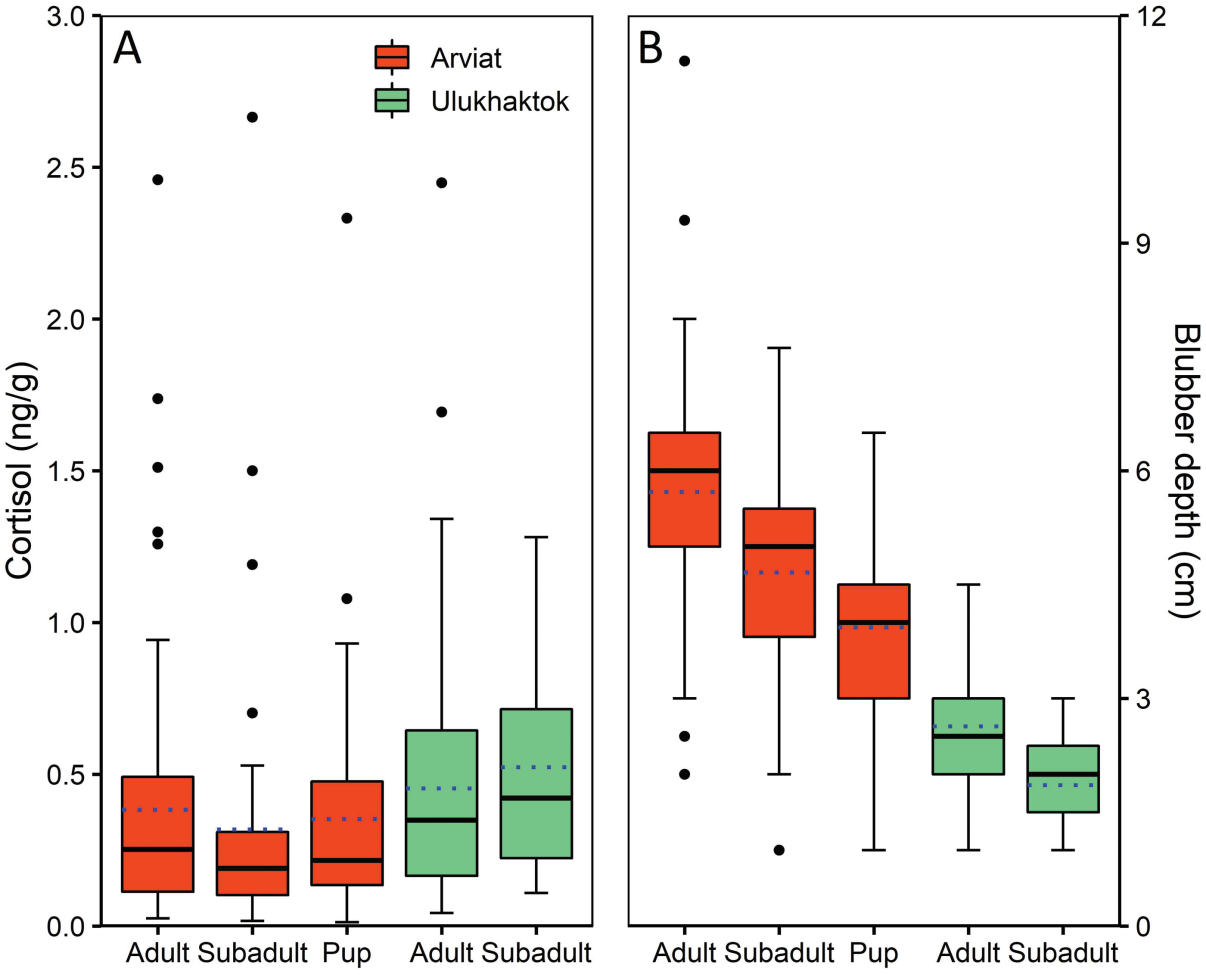
Boxplots indicating (A) blubber cortisol concentrations (ng/g) and (B) blubber depth (cm), measured at the sternum, of ringed seals sampled in Arviat, Nunavut (autumn; 2003–2012), and Ulukhaktok, Northwest Territories (spring; 2002, 2004–2005, 2007–2012). Solid horizontal lines on each boxplot indicate the median, and the dotted line represents the mean. Whiskers denote the highest and lowest points that fall within 1.5 × the interquartile range; points outside of this are outliers. The lower and upper limits of the boxes represent the first and third quartiles.

A total of 449 ringed seals were sampled from 2002 to 2012 (Table 1). For Arviat, there were no samples collected in 2002, and samples from Ulukhaktok were absent in 2003 and 2006. Overall, mean cortisol measurements were low (0.40 ± 0.03 ng/g; 0.01–2.66 ng/g), with the highest mean concentration (0.52 ± 0.12 ng/g; 0.11–1.28 ng/g) found in subadults from Ulukhaktok (Fig. 2; Table 1). In contrast, subadults from Arviat had the lowest mean concentration of cortisol (0.32 ± 0.08 ng/g; 0.02–2.66 ng/g; Fig. 2; Table 1). Log-cortisol did not differ significantly by age-class in Arviat ringed seals (ANOVA: *F*_2, 151_ = 0.98, *P* = 0.38; Table 1), or between adults and subadults from Ulukhaktok (Welch two-sample *t*-test: *t*_13.14_ = −0.95, *P* = 0.36; Table 1). Blubber depth differed significantly among age-classes for Arviat ringed seals (ANOVA: *F*_2, 260_ = 47.05, *P* < 0.0001), with post hoc tests revealing a significant increase in blubber depth with age (Tukey’s multiple comparison test: *P* < 0.001; Fig. 2; Table 1). Similarly, Ulukhaktok adults had significantly thicker blubber than subadults (Welch two-sample *t*-test: *t*_16.13_ = 4.33, *P* < 0.001; Fig. 2; Table 1). Both δ^13^C and δ^15^N differed significantly between age groups for Arviat ringed seals (Kruskal–Wallis rank-sum tests: χ^2^_2_ = 9.88, *P* < 0.01 and χ^2^_2_ = 64.65, *P* < 0.0001, respectively). The δ^13^C values in Arviat adults were significantly higher than both subadults (Dunn’s multiple comparison test: *P* = 0.03) and pups (*P* = 0.01; Table 1). For δ^15^N, all pairwise age-class comparisons differed significantly (Dunn’s multiple comparison test: *P* < 0.05; Table 1), with increases in δ^15^N with age. For Ulukhaktok ringed seals, δ^13^C did not differ significantly between adults and subadults (Welch two-sample *t*-test: *t*_10.84_ = 1.22, *P* = 0.25), but δ^15^N was significantly greater in adults than subadults (Wilcoxon rank-sum test: *W* = 1186.5, *P* < 0.0001; Table 1).

**Table 1.** Sample sizes and means (*SE*) of blubber cortisol, blubber depth, muscle δ^13^C and δ^15^N, and yearly isotopic niche breadths for ringed seals sampled in Arviat, Nunavut (autumn; 2003–2012), and Ulukhaktok, Northwest Territories (spring; 2002, 2004–2005, 2007–2012).

	Blubber cortisol		Blubber depth at sternum		Stable isotopes				Annual isotopic niches	
Age-class	*n*	(ng/g)	*n*	(cm)	*n*	δ^13^C (‰)	δ^15^N (‰)	C:N	*n*	SEA_C_ (‰^2^)
Arviat										
Adult	66	0.38 (0.05)	101	5.7 (0.1)	102	−20.42 (0.07)	+16.00 (0.08)	3.47 (0.02)	9	1.42 (0.22)
Subadult	40	0.32 (0.08)	76	4.7 (0.2)	77	−20.60 (0.09)	+15.70 (0.10)	3.48 (0.03)	9	1.65 (0.36)
Pup	48	0.35 (0.06)	86	3.9 (0.1)	87	−20.59 (0.07)	+14.87 (0.10)	3.46 (0.03)	9	1.20 (0.32)
Ulukhaktok										
Adult	101	0.45 (0.04)	141	2.6 (0.1)	126	−20.58 (0.04)	+16.72 (0.05)	3.37 (0.01)	7	0.54 (0.05)
Subadult	11	0.52 (0.12)	14	1.9 (0.2)	11	−20.81 (0.18)	+15.72 (0.25)	3.38 (0.01)	—	—

**Fig. 3.**
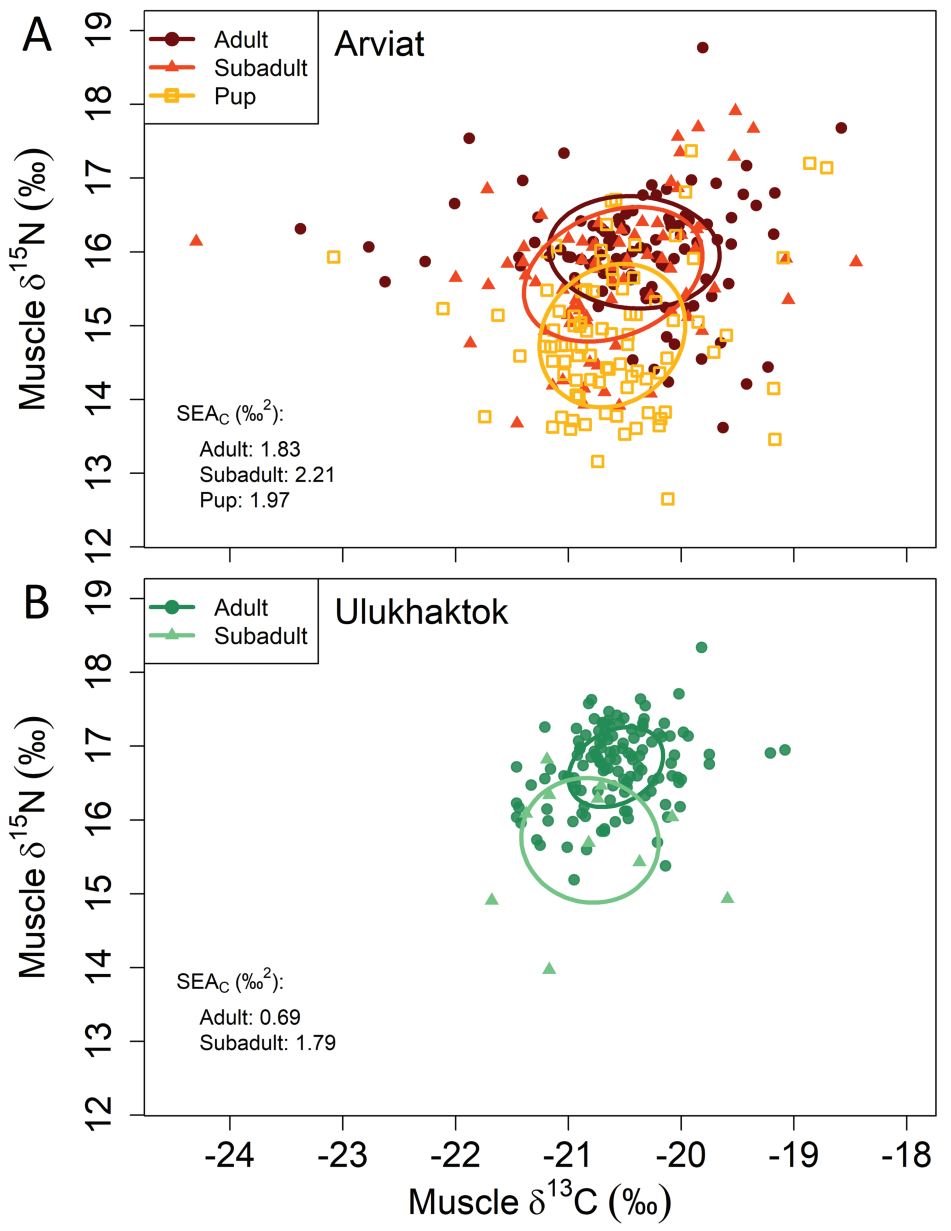
Biplots of δ^13^C, δ^15^N, and isotopic niches of ringed seal muscle sampled in (A) Arviat, Nunavut (autumn; 2003–2012), and (B) Ulukhaktok, Northwest Territories (spring; 2002, 2004–2005, 2007–2012). Standard ellipse areas, corrected for small sample sizes (SEA_C_) are denoted for each age group.

Isotopic niches were largest for Arviat subadults and smallest for Ulukhaktok adults (Fig. 3; Table 1). There was no significant difference in mean annual SEA_C_ among Arviat ringed seal age-classes (Kruskal–Wallis rank-sum test: χ^2^_2_ = 1.53, *P* = 0.47), though Arviat adults had a significantly higher mean SEA_C_ than Ulukhaktok adults (Wilcoxon rank-sum test: *W* = 58, *P* < 0.01; Table 1). We were unable to calculate and compare mean annual niches for Ulukhaktok subadults due to limited samples.

GLMMs revealed different predictive factors explaining log-cortisol concentration for both locations. The most-supported model predicting cortisol concentrations in Arviat ringed seals included age (continuous) and blubber depth, whereas blubber depth and δ^15^N best predicted cortisol concentration in Ulukhaktok ringed seals (Tables 2 and 3). For both locations, the relationship between cortisol concentration and blubber depth was negative, indicating greater cortisol levels for ringed seals with lower body condition (Table 3). For Arviat ringed seals, cortisol concentration increased with seal age, whereas, for Ulukhaktok ringed seals, cortisol concentration increased with δ^15^N (Table 3). Mean annual cortisol levels for Arviat ringed seals increased significantly with year, with a 3.4-fold increase in cortisol over the study period, from 0.14 ng/g in 2003 to 0.47 ng/g in 2012 (Table 4; best-supported model: year slope = 0.1363 ± 0.0325, *t* = 4.192, *P* < 0.01, null deviance = 7.5774 on 22 d.f., residual deviance = 4.1251 on 21 d.f.). Limited data prevented analysis of annual trends for Ulukhaktok ringed seals.

**Table 2.** Summary of general linear mixed models testing the effects of measured variables on blubber cortisol in ringed seals sampled in Arviat, Nunavut (autumn; 2003–2012), and Ulukhaktok, Northwest Territories (spring; 2002, 2004–2005, 2007–2012). Included are the ranked models, regression coefficients (intercept and factors), number of estimated parameters (d.f.), log-likelihood (logLik), Akaike’s information criterion corrected for small sample sizes (AIC_C_), ∆AIC_C_, AIC_C_ weights (Weight), and conditional *R*-squared (*R*^2^_C_) for each model. The “+” in the Sex column indicates its inclusion in the model.

Model	Intercept	Age (years)	δ^13^C (‰)	δ^15^N (‰)	Standard length (cm)	Sex	Blubber depth (cm)	d.f.	logLik	AIC_C_	ΔAIC_C_	Weight	*R* ^2^_C_
Arviat													
A_5	−1.08	0.02309					−0.1404	5	−223.461	457.3	0	0.988	0.07
A_4	−0.4482	0.02984			−0.007013		−0.124	6	−226.97	466.5	9.19	0.01	0.07
A_3	−1.394	0.03	−0.04502		−0.006804		−0.125	7	−228.046	470.9	13.54	0.001	0.06
A_2	−1.478	0.02818	−0.04984		−0.006356	+	−0.1117	9	−226.864	473	15.67	0	0.08
A_1	−1.363	0.02819	−0.04834	−0.006948	−0.006198	+	−0.1114	10	−228.043	477.7	20.32	0	0.08
Ulukhaktok													
U_5	−5.474			0.3672			−0.6808	5	−80.968	172.8	0	0.98	0.41
U_4	−6.587			0.5352	−0.01402		−0.6794	6	−83.696	180.6	7.83	0.02	0.47
U_3	−6.056	0.01137		0.5242	−0.01859		−0.6699	7	−86.731	189.2	16.34	0	0.47
U_2	−6.264	0.01107		0.5326	−0.01771	+	−0.6688	8	−87.394	193	20.19	0	0.47
U_1	−6.139	0.01106	0.005306	0.5324	−0.01781	+	−0.6689	9	−87.818	196.4	23.63	0	0.47

**Table 3.** Best-supported general linear mixed model output examining effects on the log-cortisol of ringed seals sampled in Arviat, Nunavut (autumn; 2003–2012), and Ulukhaktok, Northwest Territories (spring; 2002, 2004–2005, 2007–2012). Note that values under the *SE* column header for random effects represent standard deviation rather than standard error (*SE*), denoted by *.

Parameter	Estimate	*SE*	d.f.	*t-*value	*P*
Arviat					
(Intercept)	−1.08049	0.31161	62.23	−3.47	0.000959
Blubber depth (cm)	−0.14038	0.06628	141.7	−2.12	0.035917
Age (years)	0.02309	0.01079	143.1	2.14	0.034045
Random effects					
Year variance	0.02806	0.1675*			
Ulukhaktok					
(Intercept)	−5.4742	1.9658	70.67	−2.79	0.00687
Blubber depth (cm)	−0.6808	0.1292	70.99	−5.27	0.0000014
δ^15^N (‰)	0.3672	0.1208	70.57	3.04	0.00332
Random effects					
Year variance	0.0921	0.3035*			

**Table 4.** Summary of general linear mixed models testing the effects of measured variables on yearly mean blubber cortisol in ringed seals sampled in Arviat, Nunavut (autumn; 2003–2012). Included are the ranked models, regression coefficients (intercept and factors), number of estimated parameters (d.f.), log-likelihood (logLik), Akaike’s information criterion corrected for small sample sizes (AIC_C_), ∆AIC_C_, AIC_C_ weights (Weight), and conditional *R*-squared (*R*^2^_C_) for each model. The “+” in the Age-class column indicates its inclusion in the model.

Model	Intercept	Age-class	Standard length (cm)	SEA_C_ (‰^2^)	Blubber depth (cm)	Year	d.f.	logLik	AIC_C_	ΔAIC_C_	Weight	*R* ^2^_C_
A.Y_5	−275					0.1363	3	−12.874	33	0	0.555	0.44
A.Y_4	−273.6				0.1241	0.1353	4	−12.155	34.5	1.52	0.259	0.46
A.Y_3	−254.8		−0.01476		0.3052	0.1263	5	−11.081	35.7	2.68	0.145	0.50
A.Y_2	−244.9		−0.01328	−0.09791	0.307	0.1213	6	−10.529	38.3	5.3	0.039	0.51
A.Y_1	−233.8	+	−0.01897	−0.09287	0.28	0.1162	8	−10.425	47.1	14.13	0	0.48

## Discussion

Given the rapid climate-driven changes in the Arctic (IPCC 2014; Box et al. 2019), it is important to monitor the health of Arctic marine species to better understand changes to ­population trends. Animal health can be monitored using a variety of morphometric, chemical, and behavioral (e.g., observations of behavioral changes—lethargy, aggression, abandonment of young, altered movements/dives, etc.; Biuw et al. 2003; Huntington et al. 2017; Ostertag et al. 2018) methods. In the present study, we measured the levels of cortisol, as an indicator of chronic stress, found within the blubber of ringed seals sampled during two different seasons and in two different locations, representing ringed seals during their presumptive peak condition of the year (i.e., Arviat ringed seals) as well as at their lowest condition of the year (i.e., Ulukhaktok ringed seals; McLaren 1958; Young and Ferguson 2013). We found significant differences in blubber cortisol concentrations and body condition among locations, seasons, and age-classes. In addition, we found that body condition (blubber depth), diet (δ^13^C, δ^15^N, and isotopic niches), and morphometric and demographic factors (age, size, and sex) explained our observed patterns in cortisol concentrations.

Ringed seals sampled in Ulukhaktok during spring had higher absolute blubber cortisol concentrations than those sampled in Arviat in autumn. This can likely be explained by the timing of sampling, as Ulukhaktok ringed seals were hunted following the spring fasting, molting, and pupping period, when their body condition is at or near its lowest (McLaren 1958; Young and Ferguson 2013). A similar pattern has also been observed in harbor seals (*Phoca vitulina*) sampled during the molt (Kershaw and Hall 2016) and in western Hudson Bay polar bears sampled following the summer fast (Mislan et al. 2016). In contrast, ringed seals in Arviat were sampled following intense foraging over the summer open-water period, when their body condition is at or near its peak (McLaren 1958; Young and Ferguson 2013). Blubber thickness was generally higher for Arviat ringed seals collected in autumn than Ulukhaktok ringed seals collected in spring, which also agrees with seasonal patterns in condition. Cortisol concentrations did not differ significantly between ringed seal age-classes in Arviat or Ulukhaktok.

Blubber thickness increased significantly with age for both sampling locations/seasons. This pattern suggests that ringed seals accumulate a thicker layer of blubber as they grow, as has been observed in phocids generally (Chabot et al. 1996; Chabot and Stenson 2002; Strandberg et al. 2008). Significant increases in δ^15^N with age for both sample locations could also be related to body growth, with older/larger individuals feeding at higher trophic levels than younger individuals (Ben-David and Flaherty 2012; Yurkowski et al. 2016b). The greater diving capacity of larger seals could also allow them to access larger, deeper, and higher trophic level prey sources with higher δ^15^N (Lydersen et al. 1992; Wathne et al. 2000; Hobson et al. 2002). In contrast, higher δ^13^C values in Arviat adults than subadults and pups might suggest that adult ringed seals feed more benthically, nearshore, or on more ice-associated prey than their younger counterparts. This latter idea is supported by the tendency for adults to outcompete subadults for primary feeding and mating habitats nearshore in the fall (McLaren 1958; Krafft et al. 2007; Crawford et al. 2012), though this pattern in δ^13^C is not evident for Ulukhaktok ringed seals, possibly due to the relative lack of recent feeding, seasonal differences, or the unbalanced sample sizes between age-classes. The relatively larger isotopic niche of Arviat adults than Ulukhaktok adults could also be reflective of differences in recent feeding, as well as differences in prey availability and degree of specialization (Yurkowski et al. 2016a); however, spatial or seasonal differences in isotopic baselines (Arviat phytoplankton δ^13^C: −29.8‰, δ^15^N: +8.3‰, Graham et al. 2010; Ulukhaktok phytoplankton δ^13^C: −27.6‰, δ^15^N: +5.4‰, Roy et al. 2015) and food chain length between the two locations could also affect isotopic niche size and should be considered when interpreting our observed patterns (Vander Zanden and Fetzer 2007; Ward and McCann 2017).

For Ulukhaktok ringed seals sampled in spring, log-cortisol was best predicted by blubber depth and δ^15^N. The positive relationship between δ^15^N and cortisol suggests a possible fasting signal, which is further supported by the negative relationship to blubber depth, indicating poorer body condition (Hobson et al. 1993; Kurle and Worthy 2001; Hertz et al. 2015; Lübcker et al. 2020). Thinner ringed seals had higher δ^15^N and higher cortisol concentrations, suggesting that, as body condition decreased, ringed seals metabolized more of their own tissue, becoming enriched in ^15^N and possibly experiencing greater stress (Hobson et al. 1993; Hertz et al. 2015; Doi et al. 2017). Although a similar relationship has been found in numerous species of marine mammal (e.g., polar bears, Polischuk et al. 2001; northern fur seals *Callorhinus ursinus*, Kurle and Worthy 2001; southern elephant seals *Mirounga leonina*, Lübcker et al. 2020), other studies have shown more uncertain and variable patterns (Das et al. 2004; Gómez-Campos et al. 2011; Aguilar et al. 2014; Spurlin et al. 2019), and it has been suggested that δ^15^N is not a reliable predictor of fasting in some marine mammals, as many factors—such as gestation, lactation, and differing isoscapes between feeding locations—can confound the relationships (Gómez-Campos et al. 2011; Aguilar et al. 2014). However, given the supporting blubber measurements and the time of year Ulukhaktok ringed seals were collected in this study, a fasting signal is most likely, as fasting during the breeding season is common in some marine mammal species (Mesnick and Ralls 2009). Many female pinnipeds reduce or eliminate time spent foraging during this period, instead mobilizing stored energy to support lactation (Costa 1993; Champagne et al. 2006), whereas males may fast while competing for mates and defending territory in order to maximize reproductive success (Galimberti et al. 2007; Champagne et al. 2012a).

For Arviat ringed seals sampled in autumn, cortisol was best predicted by blubber depth and age, with cortisol increasing with age and decreasing with blubber depth; however, when testing for differences in cortisol levels among Arviat ringed seal age-classes, no significant differences were found. Given the low *R*^2^_C_ values for these models, it is possible that the effects of age on blubber cortisol are only weak, with older individuals tending to have slightly higher cortisol concentrations in their blubber compared to their younger, more naïve counterparts. As these seals are generally in their best body condition of the year (McLaren 1958; Young and Ferguson 2013), it is uncertain whether this effect is biologically significant, and additional factors not measured here (e.g., contaminants, movement patterns, food web metrics, etc.) are likely involved.

Annual mean cortisol levels of Arviat ringed seals significantly increased over the ~10-year study period, indicating that these ringed seals are possibly experiencing greater chronic stress over time. Climate change in Hudson Bay has resulted in warmer waters (Galbraith and Larouche 2011; Brand et al. 2014), a longer open-water season (Kowal et al. 2017; Andrews et al. 2018), and altered prey assemblages, evidenced by changes in the diet of predators over time (Gaston et al. 2003; Chambellant et al. 2013; Gaston and Elliott 2014). Reduced snow and ice quality has also been documented in some Arctic locations (Webster et al. 2014), impacting ringed seal pup survival (Iacozza and Ferguson 2014). These environmental changes have the potential to lead to reduced fitness of ringed seals, and, consequently, population declines. Warmer waters might challenge the physiological capabilities of ringed seals and/or their prey, leading to altered energy expenditure and intake. A longer open-water season can impact several phases of the ringed seal life cycle; for example, earlier breakup can impact ringed seal ecology by reducing the duration and spatial extent of sea-ice platforms for molting, pupping, and nursing, potentially leading to increased pup mortality. Changes to prey assemblages could lead to increased competition (Ogloff et al. 2019) for limited prey and alter dietary composition, which can have negative implications if new prey are of lower energetic quality and digestibility. Though the mechanisms are not fully understood, previous studies have already suggested linkages between climate change and reductions in ringed seal ovulation rates, pregnancy rates, and pup recruitment, as well as increased levels of stress indicators and observations of sick seals, all of which appear to have contributed to declines in abundance (Ferguson et al. 2005, 2017; Stirling 2005).

Blubber cortisol measurements in this study were low (range: 0.01–2.66 ng/g) compared to other published blubber cortisol measurements from pinnipeds. For example, cortisol concentrations in the blubber of harbor seals in Scotland ranged from 37.8 ng/g throughout the non-molting period to 1,553.6 ng/g during the molt (Kershaw and Hall 2016), and mean blubber cortisol concentrations measured in California sea lions ranged from 8.1 ± 2.1 ng/g in bycaught animals to 256.3 ± 115.7 and 235.8 ± 68.5 ng/g for dead- and live-stranded animals, respectively (Beaulieu-McCoy et al. 2017). The low cortisol values measured in the present study could be related to biological factors or to measurement error. Namely, the 60% extraction efficiency could explain low cortisol values, but this extraction efficiency is within the range of values regularly published (e.g., 30% from beluga blubber, Loseto et al. 2017; 63% from harbor seal blubber, Kershaw and Hall 2016; 77% from beluga blubber, Trana et al. 2016; 73% from humpback whale *Megaptera novaeangliae* blubber, Beaulieu-McCoy et al. 2017). Alternately, low cortisol measurements could suggest that there is little cortisol to be measured in blubber relative to other tissues. Indeed, it has been shown that blubber actively metabolizes steroid hormones in some other marine mammals (Galligan et al. 2018); however, Champagne et al. (2017) found that blubber cortisol levels can be a reliable indicator of circulating cortisol levels in bottlenose dolphins. An additional possible explanation is that, because ringed seals are well-adapted to highly variable and extreme conditions, they are resilient to stressors and are well-equipped to handle low body condition in the spring (Ferguson and Higdon 2006). For instance, they regularly perform extensive long-distance movements (Teilmann et al. 1999; Harwood et al. 2012; Hamilton et al. 2015; Ogloff et al. 2021) and energetically expensive molting and fasting phases, where they can lose up to ~60% of their body fat (McLaren 1958; Ryg et al. 1990; Young and Ferguson 2013). As such, low levels of stress during challenging conditions could be an evolved trait of ringed seals and other animals in highly variable environments, allowing them to remain sensitive and responsive to additional stressors, such as predation (Champagne et al. 2015). Similarly, low cortisol measurements have been observed in other Arctic marine mammals (Macbeth et al. 2012; Trana et al. 2016; Watt et al. 2016), lending support to this idea.

Increases in measured cortisol over time are a noteworthy trend given that the climate will likely continue to warm (IPCC 2014). Although the data here are not extensive enough to link increased cortisol levels in Arviat ringed seals with warmer conditions, further research and attention is warranted. Given that cortisol measurements were overall low, it is possible that this population of ringed seals is not presently experiencing harmful levels of stress; however, the increasing extent and number of pressures that will co-occur with warmer conditions might exacerbate acute and chronic stress levels. On an individual level, this might lead to inhibited or reduced reproduction, reduced growth, and reduced immune function (Sapolsky et al. 2000; Tilbrook et al. 2000; Sheriff et al. 2010; Dantzer et al. 2014), though these effects are not always consistent in animals (Crespi et al. 2013).

In addition, we acknowledge that cortisol measurements are not synonymous with stress and that the relationship is much more complex (Busch and Hayward 2009; MacDougall-Shackleton et al. 2019). There are many markers other than cortisol that are implicated in the stress response, including aldosterone, thyroid hormones, progesterone, and corticosteroid-binding globulin (Atkinson et al. 2015; Beaulieu-McCoy et al. 2017). Furthermore, cortisol will influence gene expression associated with numerous cellular pathways (MacDougall-Shackleton et al. 2019), and, in many animals, there are daily and seasonal fluctuations of circulating levels of cortisol unrelated to a specific response to a stressor. For instance, during winter hibernation, cortisol concentrations in black bears increase to assist with utilization of fat stores (Harlow et al. 1990). It is possible that, for ringed seals, seasonal differences in cortisol represent a biochemical adaptation to food scarcity, aiding in the mobilization of stored energy, rather than a stress response. The higher cortisol concentrations in Ulukhaktok seals support this idea, though spatial differences and other factors likely also play a role, and further investigation will be needed to draw conclusions on this matter.

We believe that pairing indicators of body condition with cortisol addresses some of the concerns with linking cortisol to stress; however, future studies incorporating other hormones and markers involved in the stress response would be beneficial and would further support our conclusions. Additional study might also address limitations with relation to sample sizes for Ulukhaktok subadults and pups as well as seasonality of sampling. The different timing of sampling precluded direct comparison among sites and seasons, but continued sampling might allow for comparisons across latitudes, seasons, and age-classes.
